# Genotypes, antibiotic resistance, and virulence diversity among *Klebsiella pneumoniae* species complex isolates in Taiwan

**DOI:** 10.1186/s13099-026-00817-5

**Published:** 2026-03-05

**Authors:** Chiung-Ju Chen, Chiao-Yu Kao, Ya-Yu Cheng, Rong-Xuan Wu, Yen-Zhen Zhang, Daniel Yeng-Fong Lin, Ming-Cheng Wang, Wei-Hung Lin, Ching-Hao Teng, Hsu-Feng Lu, Yu-Tsung Huang, Pei-Fang Tsai, Ying-Chi Li, Shih-Fen Tseng, Li-Cheng Yen, Cheng-Yen Kao

**Affiliations:** 1 Department of Pathology and Laboratory Medicine, Shin Kong Wu Ho-Su Memorial Hospital, Taipei, Taiwan; 2 Department of Biotechnology and Laboratory Science in Medicine, National Yang Ming Chiao Tung University, Taipei, Taiwan; 3 Institute of Microbiology and Immunology, College of Life Sciences, National Yang Ming Chiao Tung University, No. 155, Sec. 2, Linong Street, Taipei, 112304 Taiwan; 4 School of Medicine, College of Medicine, National Yang Ming Chiao Tung University, Taipei, Taiwan; 5 Division of Nephrology, Department of Internal Medicine, College of Medicine, National Cheng Kung University Hospital, National Cheng Kung University, Tainan, Taiwan; 6 Institute of Clinical Pharmacy and Pharmaceutical Sciences, College of Medicine, National Cheng Kung University, Tainan, Taiwan; 7 Institute of Molecular Medicine, College of Medicine, National Cheng Kung University, Tainan, Taiwan; 8 Department of Laboratory Medicine, China Medical University Hospital, Taichung, Taiwan; 9 Department of Medical Laboratory Science and Biotechnology, Asia University, Taichung, Taiwan; 10 Department of Laboratory Medicine, National Taiwan University Hospital, Taipei, Taiwan; 11 Department of Pathology, College of Medicine, National Cheng Kung University Hospital, National Cheng Kung University, Tainan, Taiwan; 12 Department of Emergency Medicine, Taoyuan General Hospital, Ministry of Health and Welfare, No.1492, Zhongshan Rd., Taoyuan Dist., 330 Taoyuan, Taiwan; 13 Health Innovation Center, National Yang Ming Chiao Tung University, Taipei, Taiwan; 14 Microbiota Research Center, National Yang Ming Chiao Tung University, Taipei, Taiwan

**Keywords:** Antibiotic resistance, *Klebsiella pneumoniae* species complex, Larvae infection, Multiplex PCR, Virulence genes

## Abstract

**Background:**

*Klebsiella pneumoniae*, *K. quasipneumoniae*, and *K. variicola* are members of the *K. pneumoniae* species complex (*Kp*SC) with distinct genotypes, virulence, and antibiotic resistance traits. However, their distribution and characteristics in Taiwan remain unknown.

**Methods:**

We evaluated 809 *Kp*SC isolates (377 from bacteremia and 432 from urinary tract infections) collected at National Cheng Kung University Hospital (NCKUH) (1999–2022), identified by multiplex PCR and 16S rDNA sequencing, and characterized by antimicrobial susceptibility testing, virulence gene profiling, capsular typing, biofilm assays, and *Galleria mellonella* larvae infection models.

**Results:**

Among the 809 *Kp*SC isolates, 752 (93.0%) were identified as *Klebsiella pneumoniae*, 37 (4.6%) as *K. quasipneumoniae*, and 20 (2.4%) as *K. variicola*. MALDI-TOF was unable to identify any of the 37 *K. quasipneumoniae* isolates correctly, but it accurately identified 17 of the 20 *K. variicola* isolates. K62 was the most prevalent capsular type among *K. pneumoniae* isolates (7/50, 14%), while K54 was the most common among *K. quasipneumoniae* isolates (5/37, 13.5%). Among the 20 *K. variicola* isolates, K35 and K49 were the major capsular serotypes, each represented by three isolates (3/20, 15%). Notably, 16.8% of *Kp*SC isolates could not be assigned to any serotype based on *wzi* sequencing. The prevalence of *iucA*, *iroB*, *irp1*, *irp2*, _*p*_*rmpA*, _*p*_*rmpA2*, and *peg-344* was significantly higher in *K. pneumoniae* than in *K. quasipneumoniae* and *K. variicola*. In contrast, only *kfuBC* was more common in *K. variicola* than in *K. pneumoniae* and *K. quasipneumoniae*. The virulence score was highest in *K. pneumoniae* (6.2 genes), followed by *K. quasipneumoniae* (4.6 genes) and *K. variicola* (4.4 genes) (*p* = 0.002). *K. pneumoniae* demonstrated significantly higher resistance rates to gentamicin (*p* = 0.005), ciprofloxacin (*p* = 0.013), levofloxacin (*p* = 0.003), and sulfamethoxazole/trimethoprim (*p* = 0.036), compared to *K. quasipneumoniae* and *K. variicola*. The biofilm formation capacity of *K. pneumoniae* was found to be higher than that of *K. quasipneumoniae* and *K. variicola*. However, on day seven, the survival rate of larvae infected with *K. pneumoniae* was 25.1%, while only 9.7% and 8.5% of larvae survived in *K. quasipneumoniae* and *K. variicola*, respectively (*p* < 0.0001).

**Conclusion:**

*K. pneumoniae*, *K. quasipneumoniae*, and *K. variicola* in Taiwan exhibit distinct genotypic and phenotypic characteristics. *K. quasipneumoniae* and *K. variicola* displayed higher mortality in larvae infection model, underscoring the importance of continued surveillance of their dissemination.

## Introduction

*Klebsiella*, a genus of gram-negative bacteria within the *Enterobacteriaceae* family, is recognized for causing a broad spectrum of clinical infections, including pneumonia, intra-abdominal clinical infections, urinary tract infections (UTIs), bloodstream clinical infections, and liver abscesses, especially in people with diabetes [1]. The rise of multidrug-resistant (MDR) *Klebsiella* strains has escalated this pathogen to a significant global public health concern [2, 3]. The *Klebsiella pneumoniae* species complex (*Kp*SC) has been classified into several phylogroups: Kp1 through Kp7. Kp1 corresponds to *K. pneumoniae*, Kp2 includes *K. quasipneumoniae* subsp. *quasipneumoniae*, Kp3 comprises *K. variicola* subsp. *variicola*, Kp4 contains *K. quasipneumoniae* subsp. *similipneumoniae*, Kp5 and Kp6 have been associated with *K. variicola* subsp. *tropica* and *K. quasivariicola*, respectively, and Kp7 has been linked to *K. africana* [4–7]. Among these, *K. variicola* subsp. *variicola* (Kp3) and *K. quasipneumoniae* (Kp2 and Kp4) are increasingly recognized as emerging human pathogens [8, 9]. In contrast, *K. quasivariicola* (Kp6) has been documented in only a limited number of clinical cases [10], and *K. africana* (Kp7) has been reported in a single clinical case [4].

Both *K. quasipneumoniae* and *K. variicola* have been identified as carriers of extended-spectrum β-lactamase (ESBL) and carbapenemase-producing genes [9, 11]. Additionally, *K. variicola* is often associated with healthcare-associated infections and has been reported to cause more severe invasive disease and poorer clinical outcomes compared to *K. pneumoniae* [12]. *K. quasipneumoniae* has two subspecies, among which *K. quasipneumoniae* subsp. *similipneumoniae* has been identified as a pathogen causing neonatal septicemia in China and has also caused an outbreak of neonatal septicemia in a tertiary hospital in Nigeria [13, 14]. It can also exhibit a hypervirulent phenotype leading to liver abscesses [15]. Studies have indicated that the prognosis for *K. quasipneumoniae* infections is worse than that for *K. pneumoniae*, despite having fewer virulence genes [7, 15, 16].

The iron-uptake system is crucial for bacterial proliferation; relevant genes include *entB*, *ybtS*, *iutA*, and *kfu* (siderophore biosynthesis and iron receptor/transport genes). Studies have shown that *K. variicola* carries a higher proportion of the *kfu* gene than *K. pneumoniae* and *K. quasipneumoniae*, while *K. pneumoniae* has a higher prevalence of the *iutA* and *ybtS* genes. On the other hand, *K. quasipneumoniae* has fewer iron uptake-associated genes but a higher proportion of genes related to allantoin metabolism, such as *allS* [6]. However, the prevalence of these genes may vary among isolates from different regions [17], and no such data are yet available from Taiwan. Moreover, the direct association between the prevalence of these genes and bacterial pathogenesis remains unclear.

Previous studies have demonstrated that multiplex PCR targeting species-specific markers can accurately distinguish *K. pneumoniae*, *K. quasipneumoniae*, and *K. variicola*, serving as a reliable reference for subsequent identification methods, including MALDI-TOF. While MALDI-TOF can correctly identify *K. pneumoniae* and *K. variicola*, it may misclassify *K. quasipneumoniae* depending on the instrument’s database [18]. Similarly, 16S rDNA sequencing is limited in differentiating species within the *Kp*SC due to high sequence similarity (> 99%). Recently, Ohno et al. used multiplex PCR analysis to identify 178 *K. pneumoniae* isolates, 60 *K. variicola* isolates, and 14 *K. quasipneumoniae* isolates. However, MALDI-TOF results showed that among the 14 isolates identified as *K. quasipneumoniae* by multiplex PCR, only one was confirmed as *K. quasipneumoniae*, whereas for the 60 *K. variicola* isolates, 58 were still identified as *K. variicola* by MALDI-TOF [19]. In contrast, whole-genome sequencing and the Klebsiella MALDI typeR database accurately identified all three species and their subspecies [20].

The phylogenetic and phenotypic diversity within the *Kp*SC underscores the complexity of accurately identifying its members and understanding their distinct pathogenic potentials, an essential step toward effective clinical management and epidemiological surveillance. However, most previous studies have focused primarily on *K. pneumoniae*, with scarce data available on the epidemiological, genotypic, and phenotypic characteristics of *K. quasipneumoniae* and *K. variicola*, especially in Taiwan. In this study, we first systematically compared *K. pneumoniae*, *K. quasipneumoniae*, and *K. variicola* isolates in Taiwan regarding their genotypic and phenotypic profiles, including virulence, antimicrobial resistance, and biofilm formation.

## Materials and methods

### *Klebsiella pneumoniae* collection

This study was approved by the Institutional Review Board of National Cheng Kung University Hospital (NCKUH) (approved number: A-ER-112–213). NCKUH is located in the North District of Tainan in southern Taiwan, is a medical center and teaching hospital affiliated with National Cheng Kung University, with a total capacity of approximately 1,342 beds. A total of 809 *Kp*SC isolates (377 from bacteremia patients and 432 from UTI patients) were retrospectively collected at NCKUH from 1999 to 2022. All *Kp*SC isolates were stored at −80 °C in lysogenic broth (LB) with 20% glycerol (v/v). *Kp*SC isolates were either grown in LB or on LB agar for testing.

### *K. pneumoniae*, *K. quasipneumoniae*, and *K. variicola* identification by multiplex PCR, 16S rDNA sequencing, and MALDI-TOF

Multiplex PCR was conducted for initial species identification, with primers and PCR conditions to detect phosphoenolpyruvate mutase, *tctA*, and diguanylate phosphodiesterase, following previously published literature [21]. *Kp*SC isolates were further validated by using 16S rDNA sequence analysis as previously described [22]. *K. pneumoniae*, *K. variicola*, and *K. quasipneumoniae* isolates were also detected using the Bruker Biotyper (version 2.0) MALDI-TOF system, following the manufacturer’s guidelines.

### Capsular type determination by PCR and *wzi* sequencing

Genomic DNA from the isolates was extracted using a boiling method and evaluated by PCR to detect capsular serotype-specific genes, including K1, K2, K5, K20, K47, K54, K57, and K64, following previous studies [23]. The *wzi* sequencing and the analysis of the corresponding K types were conducted with reference to previously published studies [24].

### Multi-locus sequence typing of *K. varricola*

Multilocus sequence typing (MLST) of *K. variicola* was performed by PCR amplification and sequencing of seven housekeeping genes (*leuS*, *pgi*, *pgk*, *phoE*, *pyrG*, *rpoB*, and *fusA*) following the protocol described in a previous study [25]. MLST allele assignments and sequence type determination were conducted by comparing the obtained sequences against the *K. variicola* MLST database (https://mlstkv.insp.mx/).

### Detection of virulence-associated genes

Nineteen virulence-associated genes were detected by PCR using the GeneExplorer thermal cycler (Bioer Technology, China) with Fast-Run™ 2× Taq Master Mix (Protech, Taipei, Taiwan) and 96-well PCR plates (MB-P96-T and MB-PSM, Gunster Biotech, Taiwan). The primer sequences and PCR conditions have been described in our previous publication [26]. The targeted genes included those involved in iron acquisition systems (*entB*, *iucA*, *irp1*, *irp2*, *iroB*, *kfuBC*, *ybtA*, *ybtS*), capsule synthesis (_*p*_*rmpA*, _*p*_*rmpA2*, *wcaG*), metabolite transport (*peg-344*, *peg-589*, *peg-1631*), proteolysis (*htrA*), adhesion (*fimH*, *mrkD*), LPS biosynthesis (*wabG*), and allantoin metabolism (*allS*).

### Mucoid phenotype determination

The string test associated with the hypermucoviscosity of *Kp*SC was conducted according to a previous study [27]. The criterion for a positive string test result was a mucoid string > 5 mm in length.

### Antibiotic susceptibility testing

Antibiotic susceptibility to 20 agents was assessed using standard disk diffusion methods according to the Clinical and Laboratory Standards Institute (CLSI) guidelines. The agents tested included amikacin (30 µg), amoxicillin/clavulanic acid (20/10 µg), ampicillin (10 µg), ampicillin/sulbactam (10/10 µg), cefazolin (30 µg), cefmetazole (30 µg), cefoxitin (30 µg), ceftazidime (30 µg), ceftriaxone (30 µg), ciprofloxacin (5 µg), colistin (10 µg), ertapenem (10 µg), gentamicin (10 µg), imipenem (10 µg), meropenem (10 µg), levofloxacin (5 µg), piperacillin/tazobactam (100/10 µg), sulfamethoxazole/trimethoprim (23.75/1.25 µg), tetracycline (30 µg), and tigecycline (15 µg) (BD BBL Sensi-Disc; Becton, Dickinson and Company, MD, USA). *E. coli* ATCC 25,922 was used as a quality control strain. Susceptibility was interpreted according to the CLSI guidelines (34th edition, version 2024), with isolates classified as MDR (non-susceptible to at least one agent in three or more categories), extensively drug-resistant (XDR; non-susceptible to at least one agent in all but two or fewer categories), or pandrug-resistant (PDR; non-susceptible to all agents tested) [28]. For tigecycline disk diffusion testing, a zone of ≥ 18 mm was considered sensitive while ≤ 15 mm was considered resistant, according to the breakpoint tables for interpretation of MICs and zone diameters in European Committee on Antimicrobial Susceptibility Testing (EUCAST) (Version 6.0, 2016). Isolates with a colistin disk zone diameter of ≥ 14 mm were classified as susceptible, while those with a diameter of ≤ 11 mm were considered resistant [29].

### ESBL producer detection

Phenotypic detection of ESBL production was performed using the CLSI-recommended combined disk test. Briefly, bacterial suspensions were prepared to a 0.5 McFarland turbidity standard and inoculated uniformly onto Mueller–Hinton agar plates. Ceftazidime (30 µg) and ceftazidime–clavulanate (30/10 µg) disks were placed on the agar surface, and plates were incubated at 35 °C for 16–18 h. An isolate was considered ESBL-positive when the inhibition zone diameter around the ceftazidime–clavulanate disk was ≥ 5 mm larger than that around the ceftazidime disk alone.

### Biofilm formation assay

Biofilm formation was assessed using a 96-well flat-bottom polystyrene microtiter plate, with slight modifications to previously described methods [30]. A total of 20 µL of overnight bacterial culture was inoculated into 180 µL of LB or M9 medium in a 96-well plate, followed by incubation at 37 °C with 5% CO₂ for 24, 48, and 72 h. After incubation, the supernatant was removed, and biofilms were fixed with 200 µL methanol for 30 min. The methanol was then discarded, and the biofilms were stained with 0.1% crystal violet for 30 min. Wells were washed with PBS, and the bound crystal violet was eluted with 95% ethanol for 30 min. Biofilm formation was quantified by measuring absorbance at 590 nm.

### *Galleria mellonella* larvae infection

The larvae infection model was used to determine the virulence of 107 *Kp*SC isolates, following our previous study [31]. Larvae weighing greater than 0.24 g were used for this assay. The larvae were injected with 10 µL of inoculum (total 2.5 × 10⁶ CFU) into the reduced left proleg, while larvae injected with the same volume of PBS served as negative controls. The virulence of each isolate was tested in 5 larvae, and the survival of the larvae was monitored every 24 h for seven days after injection while they were incubated in Petri dishes at 37 °C under normal aerobic conditions. Larvae were considered dead if they did not respond to gentle poking with a pipette tip. To ensure reproducibility, the larvae infection assay was conducted in biological duplicates (10 larvae per isolate). Larvae injected with PBS served as the negative control, while those inoculated with the hypervirulent *K. pneumoniae* strain NTUH-K2044 [32] were used as the positive control in this experiment.

### Statistical analysis

Categorical variables were compared using chi-square tests, and statistical analyses were performed using SPSS version 22.0 (IBM, Armonk, NY, USA). Statistical analyses of biofilm formation and larvae survival proportions were carried out using GraphPad Prism (version 10.0.2, USA). All results are presented as mean ± standard deviation. Group differences were assessed using unpaired t-test, while larvae survival was compared using the log-rank (Mantel-Cox) test. A *p*-value of < 0.05 indicated statistical significance.

## Results

### The prevalence of *K. pneumoniae*, *K. quasipneumoniae*, and *K. variicola* across different sample sources and collection periods

Initially, multiplex PCR was used for species identification, and among the 809 *Kp*SC isolates, 749 (92.6%) were identified as *K. pneumoniae*, 37 (4.6%) as *K. quasipneumoniae*, and 23 (2.8%) as *K. variicola*. Due to the large number of *K. pneumoniae* isolates, we selected 10 *K. pneumoniae* isolates from each year/period in Table 1, including 5 from blood and 5 from urine. The selection was made by picking one isolate every 10 numbers, for a total of five isolates. For blood isolates from 2019 to 2022, we selected isolates at intervals of every five isolates. Therefore, to further validate species identification, 50 *K. pneumoniae* isolates along with the 23 *K. variicola* and 37 *K. quasipneumoniae* isolates identified by multiplex PCR using 16S rDNA sequencing. The sequencing results confirmed the multiplex PCR findings for *K. pneumoniae* and *K. quasipneumoniae*, but revealed that three *K. variicola* isolates were *K. pneumoniae*. Therefore, we identified 752 *K. pneumoniae*, 37 *K. quasipneumoniae*, and 20 *K. variicola* (Table 1). Moreover, MALDI-TOF failed to identify all 37 *K. quasipneumoniae* isolates, but accurately identified 17 of the 20 *K. variicola* isolates. *K. quasipneumoniae* accounted for 3.2%−5.7% of blood samples and 1.6%−7.5% of urine samples over five collection periods. Similarly, *K. variicola* comprised 1.2%−5.7% of blood samples and 0%−3.3% of urine samples during the same periods (Table 1).

**Table 1:** Collection years, sample sources, and species distribution of 809 *Klebsiella pneumoniae* species complex isolates

`	Year of collection, *n* (%)										
	1999		2004		2009		2014		2019–2022		
Species	Blood	Urine	Blood	Urine	Blood	Urine	Blood	Urine	Blood	Urine	Total
***K. pneumoniae***	87 (93.6)	58 (95.1)	78 (88.6)	79 (92.9)	76 (95.0)	96 (97.0)	84 (92.3)	88 (93.6)	23 (92.0)	83 (89.2)	752 (93.0)
***K. quasipneumoniae***	3 (3.2)	1 (1.6)	5 (5.7)	6 (7.1)	3 (3.8)	3 (3.0)	5 (5.5)	3 (3.2)	1 (4.0)	7 (7.5)	37 (4.6)
***K. variicola***	3 (3.2)	2 (3.3)	5 (5.7)	0 (0)	1 (1.2)	0 (0)	2 (2.2)	3 (3.2)	1 (4.0)	3 (3.3)	20 (2.4)
**Total**	93	61	88	85	80	99	91	94	25	93	809

### The distribution of capsular types of different species of *Kp*SC

We further evaluated the distribution of capsular types by PCR to determine their association with different *Kp*SC species. Among the 107 *Kp*SC isolates evaluated, K54 was the most prevalent (7 isolates, 6.5%), followed by K2 (5 isolates, 4.7%) and K1 (4 isolates, 3.7%). In particular, 77.6% of the *Kp*SC isolates (83 isolates) were not classified into any of the eight serotypes detected by PCR. Therefore, we performed *wzi* sequencing analysis, and the results are presented in Table 2. *Kp*SC isolates exhibited highly diverse capsular types, but only 9 *K. pneumoniae* isolates, 7 *K. quasipneumoniae* isolates, and 2 *K. variicola* isolates could not have their capsular types determined based on *wzi* sequencing or showed the possibility of having more than one type (18 out of 107 *Kp*SC strains, 16.8%). K62 and K54 were the most common capsular types in *K. pneumoniae* and *K. quasipneumoniae*, respectively, while K35 and K49 were the most common in *K. variicola* (Table 2).

**Table 2:** Distribution of capsule serotypes of *K. pneumoniae*, *K. quasipneumoniae*, and *K. variicola* in *Klebsiella pneumoniae* species complex

Capsule type	Klebsiella pneumoniae species complex, *n* (%)			
	K. pneumoniae (*n* = 50)	K. quasipneumoniae (*n* = 37)	K. variicola (*n* = 20)	Total (*n* = 107)
K1	2 (4.0)	2 (8.1)	0	4 (3.7)
K2	5 (10.0)	0	0	5 (4.7)
K3	2 (4.0)	0	0	2 (1.9)
K5	0 (4.0)	1 (2.7)	0	1 (0.9)
K7	1 (2.0)	0	0	1 (0.9)
K9	1 (2.0)	0	0	1 (0.9)
K10	2 (4.0)	0	1 (5.0)	3 (2.8)
K11	1 (2.0)	0	0	1 (0.9)
K14	1 (2.0)	1 (2.7)	0	2 (1.9)
K17	2 (4.0)	1 (2.7)	0	3 (2.8)
K19	0	1 (2.7)	0	1 (0.9)
K20	1 (2.0)	0	0	1 (0.9)
K24	3 (6.0)	2 (8.1)	1 (5.0)	6 (5.6)
K25	0	0	1 (5.0)	1 (0.9)
K27	2 (4.0)	1 (2.7)	2 (10.0)	5 (4.7)
K28	1 (2.0)	0	0	1 (0.9)
K30	0	1 (2.7)	0	1 (0.9)
K31	3 (6.0)	0	1 (5.0)	4 (3.7)
K34	1 (2.0)	0	0	1 (0.9)
K35	0	2 (8.1)	3 (15.0)	5 (4.7)
K38	1 (2.0)	0	0	1 (0.9)
K39	0	1 (2.7)	0	1 (0.9)
K45	1 (2.0)	0	0	1 (0.9)
K47	2 (4.0)	0	0	2 (1.9)
K48	0	2 (8.1)	1 (5.0)	3 (2.8)
K49	0	4 (10.8)	3 (15.0)	7 (6.5)
K52	0	1 (2.7)	0	1 (0.9)
K53	0	0	1 (5.0)	1 (0.9)
K54	0	5 (13.5)	2 (10.0)	7 (6.5)
K57	0	1 (2.7)	1 (5.0)	2 (1.9)
K58	0	0	1 (5.0)	1 (0.9)
K60	0	3 (8.1)	0	3 (2.8)
K62	7 (14.0)	0	0	7 (6.5)
K64	2 (4.0)	0	0	2 (1.9)
K81	0	1 (2.7)	0	1 (0.9)
Others^a^	9 (18.0)	7 (18.9)	2 (10.0)	18 (16.8)

^a^Others refers to isolates that either possess capsular types not listed in the table or show the possibility of having more than one capsular types based on *wzi* analysis

### MLST types of *K. varricola*

Due to the large number of isolates and associated costs, MLST typing was performed for 20 *K. variicola* isolates. Of these, only five isolates could be successfully typed, corresponding to ST10, ST142, ST235, ST335, and ST750 (one isolate each). For the *pgk* and *pyrG* loci, nine and two isolates, respectively, either failed to produce PCR products or lacked matching sequences in the database, suggesting the presence of potentially novel MLST types.

### The prevalence of virulence-associated genes, virulence score, and string test positivity are associated with *Kp*SC members

Previous studies have shown differences in the distribution of virulence factors among *K. pneumoniae*, *K. quasipneumoniae*, and *K. variicola*. Therefore, we evaluated 19 genes associated with virulence, virulence scores, and the positivity rate of the string test in relation to these three species (Table 3). Among iron acquisition-related genes, *entB*, *ybtS*, and *ybtA* did not show significant differences in prevalence between species (*p* = 0.204, 0.948, and 0.093, respectively). The prevalence of *iucA*, *iroB*, *irp1*, and *irp2* was higher in *K. pneumoniae* than in *K. quasipneumoniae* and *K. variicola*. Additionally, *kfuBC* was detected in 45% of *K. variicola* isolates but in only 12% of *K. pneumoniae* and 8.1% of *K. quasipneumoniae* (*p* = 0.001).

**Table 3:** Distribution of 19 virulence-associated genes, virulence score, and string test-positive of *K. pneumoniae*, *K. quasipneumoniae*, and *K. variicola* in *Klebsiella pneumoniae* species complex

Virulence genes and phenotypes	Klebsiella pneumoniae species complex, *n* (%)				
	K. pneumoniae (*n* = 50)	K. quasipneumoniae (*n* = 37)	K. variicola (*n* = 20)	Total	*p* value
Iron-acquisition					
*iucA*	14 (28.0)	2 (5.4)	1 (5.0)	17 (15.9)	**0.006**
*iroB*	11 (22.0)	2 (5.4)	0 (0)	13 (12.1)	**0.012**
*entB*	49 (98.0)	33 (89.2)	18 (90.0)	100 (93.4)	0.204
*irp1*	13 (26.0)	2 (5.4)	0 (0)	15 (14.0)	**0.003**
*irp2*	22 (44.0)	9 (24.3)	3 (15.0)	34 (31.8)	**0.030**
*ybts*	9 (18.0)	6 (16.2)	3 (15.0)	18 (16.8)	0.948
*kfuBC*	6 (12.0)	3 (8.1)	9 (45.0)	18 (16.8)	**0.001**
*ybtA*	17 (34.0)	8 (21.6)	2 (10.0)	27 (25.2)	0.093
**Hypermucoviscosity**					
_*p*_ *rmpA*	11 (22.0)	2 (5.4)	0 (0)	13 (12.1)	**0.012**
_*p*_ *rmpA2*	12 (24.0)	2 (5.4)	1 (5.0)	15 (14.0)	**0.021**
**Capsule-associated**					
*wcaG*	2 (4.0)	5 (13.5)	1 (5.0)	8 (7.5)	0.223
**Adhesins**					
*mrkD*	44 (88.0)	32 (86.5)	19 (95.0)	95 (88.8)	0.606
**Others**					
*peg-344*	10 (20.0)	2 (5.4)	0 (0)	12 (11.2)	**0.022**
*peg-589*	13 (26.0)	6 (16.2)	2 (10.0)	21 (19.6)	0.255
*peg-1631*	13 (26.0)	8 (21.6)	2 (10.0)	23 (21.5)	0.338
*htrA*	6 (12.0)	4 (10.8)	2 (10.0)	12 (11.2)	0.967
*allS*	10 (20.0)	8 (21.6)	4 (20.0)	22 (20.6)	0.981
**Virulence score** ^a^	6.2 (3.0)	4.6 (1.8)	4.4 (1.5)	5.3 (2.6)	0.241
**String test positive**	6 (12.0)	0 (0)	0 (0)	6 (5.6)	**0.027**

^a^Virulence score is present as mean ± SD. We did not detect any isolates carrying the adhesin *fimH* gene, and all isolates possessed the *wabG* gene

For hypermucoviscosity-associated genes, _*p*_*rmpA* and _*p*_*rmpA2* were significantly more prevalent in *K. pneumoniae* than in *K. quasipneumoniae* and *K. variicola* (*p* = 0.012 and 0.021, respectively). In contrast, *wabG*, which is involved in LPS biosynthesis, was present in all *K. pneumoniae*, *K. quasipneumoniae*, and *K. variicola* isolates. The capsule-associated gene *wcaG* was slightly more prevalent in *K. quasipneumoniae* isolates (13.5%) than in *K. pneumoniae* (4%) and *K. variicola* (5%) isolates (*p* = 0.223). The adhesion-related gene *fimH* was not detected in any of the 107 *Kp*SC isolates, whereas *mrkD* showed a high prevalence across *K. pneumoniae*, *K. quasipneumoniae*, and *K. variicola* isolates, with rates of 88%, 86.5%, and 95%, respectively (*p* = 0.606).

Additionally, *peg-344* was more prevalent in *K. pneumoniae* than in *K. quasipneumoniae* and *K. variicola* (*p* = 0.022), while *peg-589* and *peg-1631* were slightly more prevalent in *K. pneumoniae* and *K. quasipneumoniae* than in *K. variicola* (*p* = 0.255 and 0.338, respectively). The total number of virulence genes per isolate, defined as the virulence score, was highest in *K. pneumoniae* (6.2 genes), followed by *K. quasipneumoniae* (4.6 genes) and *K. variicola* (4.4 genes) (*p* = 0.002). Furthermore, only 12% of the *K. pneumoniae* isolates were positive for the string test, while all *K. quasipneumoniae* and *K. variicola* isolates were negative (*p* = 0.027).

Overall, nine *K. pneumoniae* isolates and two *K. quasipneumoniae* isolates carried the five hypervirulence-associated genes: _*p*_*rmpA*, _*p*_*rmpA2*, *iroB*, *iucA*, and *peg-344*. Among them, six *K. pneumoniae* isolates also exhibited a positive string test, indicating the hypermucoviscosity phenotype. These included isolates of K1, K2 (2 isolates), K34, K64, and one isolate with an unidentified capsular type. In contrast, the three string test–negative *K. pneumoniae* isolates belonged to K1, K2, and K24. For *K. quasipneumoniae*, although both isolates carried all five hypervirulence-associated genes, they were string test–negative, corresponding to K19 and K48, respectively. No *K. variicola* isolate was found to carry all five of these virulence genes simultaneously.

### *K. pneumoniae* exhibited higher antibiotic resistance compared to *K. quasipneumoniae* and *K. variicola*

In the antibiotic susceptibility analysis, no imipenem-resistant or pandrug-resistant isolates were identified in 107 *Kp*SC isolates. However, *K. pneumoniae* exhibited a higher resistance rate to gentamicin (42%) compared to *K. quasipneumoniae* (13.5%) and *K. variicola* (15%) (*p* = 0.005) (Table 4). *K. pneumoniae* also demonstrated significantly higher resistance to fluoroquinolones, including ciprofloxacin and levofloxacin, than *K. quasipneumoniae* and *K. variicola* (*p* = 0.013 and 0.003, respectively). Additionally, 42% of *K. pneumoniae* isolates were resistant to sulfamethoxazole/trimethoprim, while resistance rates for *K. quasipneumoniae* and *K. variicola* were 32.4% and 10%, respectively (*p* = 0.036). Overall, *K. pneumoniae* demonstrated higher antibiotic resistance, with 62% classified as XDR, compared to 45.9% of *K. quasipneumoniae* and 25% of *K. variicola* isolates were XDR isolates (*p* = 0.017). In addition, four *K. pneumoniae* (4/50, 8%), six *K. quasipneumoniae* (6/37, 16%), and three *K. variicola* (3/20, 15%) isolates were identified as ESBL producers. The distribution of ESBL producers among the three species did not show a statistically significant difference (*p* = 0.465).

**Table 4:** Distribution of antibiotic non-susceptible isolates of *K. pneumoniae*, *K. quasipneumoniae*, and *K. variicola* in *Klebsiella pneumoniae* species complex

Antibiotics and phenotype^a^	Klebsiella pneumoniae species complex, *n* (%)					
	K. pneumoniae (*n* = 50)	K. quasipneumoniae (*n* = 37)	K. variicola (*n* = 20)	Total		*p* value
**Aminoglycoside**						
AN	5 (10.0)	2 (5.4)	0 (0)	7 (6.5)		0.293
GM	21 (42.0)	5 (13.5)	3 (15.0)	29 (27.1)		**0.005**
**Penicillins**						
AM	50 (100.0)	36 (97.3)	20 (100.0)	106 (99.1)		0.385
AMC	19 (38.0)	11 (29.7)	3 (15.0)	33 (30.8)		0.167
**Penicillins + β-lactamase inhibitors**						
SAM	20 (40.0)	10 (27.0)	5 (25.0)	35 (32.7)		0.318
TZP	6 (12.0)	3 (8.1)	3 (15.0)	12 (11.2)		0.713
**Carbapenems**						
ETP	0 (0)	2 (5.4)	0 (0)	2 (1.9)		0.145
MEM	0 (0)	1 (2.7)	0 (0)	1 (0.9)		0.385
**Non-extended-spectrum cephalosporins**						
CZ	19 (38.0)	16 (43.2)	4 (20.0)	39 (36.4)		0.210
CMZ	8 (16.0)	6 (16.2)	1 (5.0)	15 (14.0)		0.436
**Extended-spectrum cephalosporins**						
CRO	8 (16.0)	10 (27.0)	3 (15.0)	21 (19.6)		0.373
CAZ	13 (26.0)	11 (29.7)	3 (15.0)	27 (25.2)		0.467
**Cephamycins**						
FOX	9 (18.0)	6 (16.2)	2 (10.0)	17 (15.9)		0.709
**Fluoroquinolones**						
CIP	24 (48.0)	8 (21.6)	4 (20.0)	36 (33.6)		**0.013**
LVX	19 (38.0)	3 (8.1)	3 (15.0)	25 (23.4)		**0.003**
**Tetracyclines**						
TE	23 (46.0)	13 (35.1)	4 (20.0)	40 (37.4)		0.120
**Glycylcyclines**						
TIG	1 (2.0)	1 (2.7)	0 (0)	2 (1.9)		0.769
**Folate pathway inhibitors**						
SXT	21 (42.0)	12 (32.4)	2 (10.0)	35 (32.7)		**0.036**
**Polymyxins**						
CL	0 (0)	1 (2.7)	0 (0)	1 (0.9)		0.385
**Phenotype (MDR/XDR)**	19 (38.0)/ 31 (62.0)	20 (54.1)/ 17 (45.9)	15 (75.0)/ 5 (25.0)	54 (50.5)/ 53 (49.5)		**0.017**
**ESBL**	4 (8.0)	6 (16.2)	3 (15.0)	13 (12.1)		0.465

### *K. pneumoniae* exhibited higher biofilm production in both M9 and LB media compared to *K. quasipneumoniae* and *K. variicola*

**Fig. 1:**
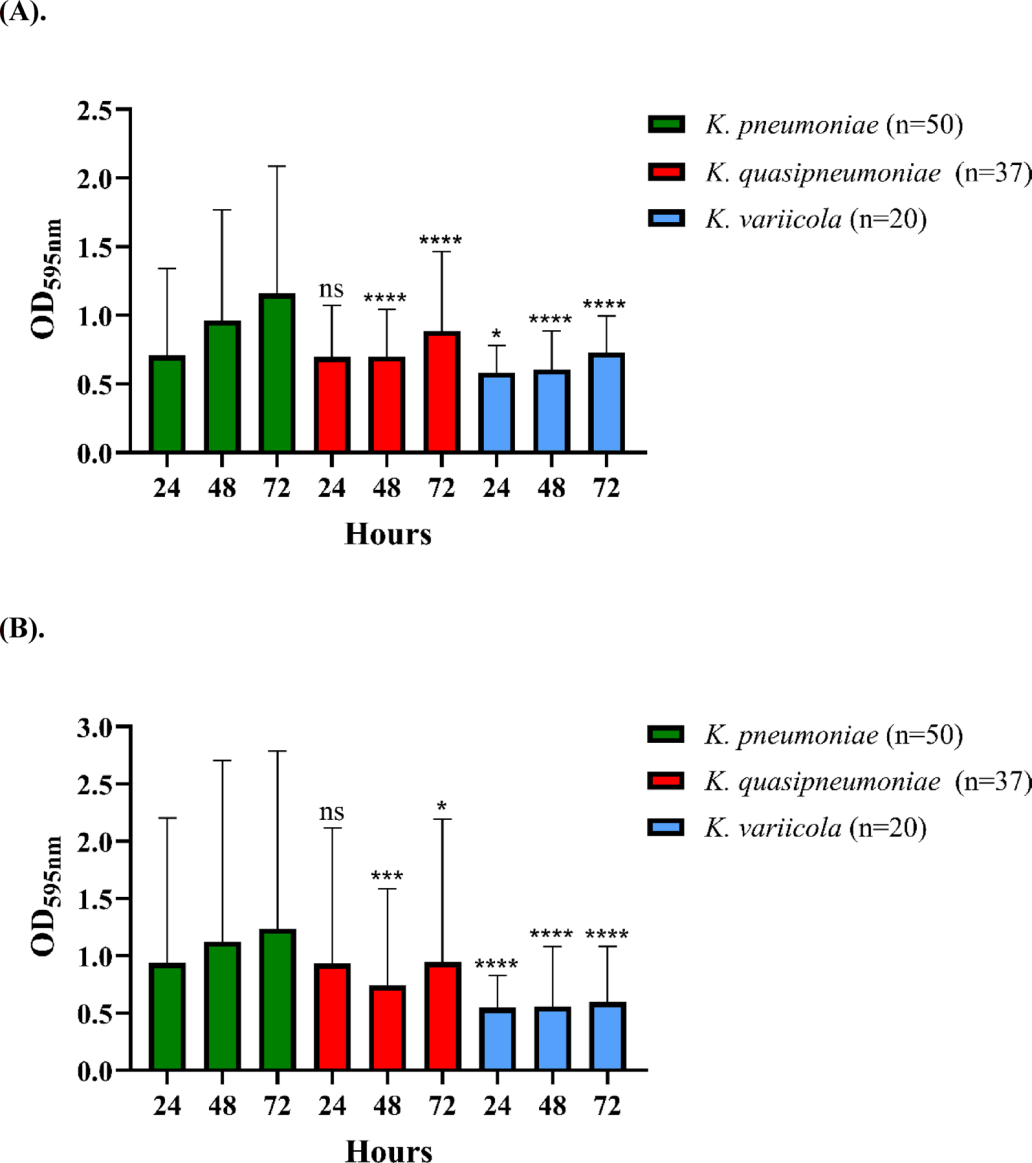
The biofilm formation of *Kp*SC isolates in different species was assessed in M9 (**A**) and LB (**B**) media. Bacteria were incubated for 24, 48, and 72 h, and biofilm production was measured. Data represent the averages of three independent experiments conducted in technical replicates. Results are expressed as mean ± standard deviation. Statistical comparisons were conducted between each species and *K. pneumoniae* at the same incubation time. ns, not significant; *, *p* < 0.05; ***, *p* < 0.001; ****, *p* < 0.0001

Biofilm formation is associated with bacterial pathogenicity; therefore, we evaluated the biofilm production of 107 *Kp*SC isolates in minimal medium (M9) and nutrient-rich medium (LB) after 24, 48, and 72 h of incubation (Fig. 1). Overall, the 50 *K. pneumoniae* isolates exhibited slightly higher biofilm formation than the 37 *K. quasipneumoniae* and 20 *K. variicola* isolates. In M9 medium, *K. pneumoniae* produced significantly more biofilm than *K. quasipneumoniae* and *K. variicola* after 48 and 72 h of incubation (*p* < 0.0001). However, after 24 h, *K. pneumoniae* exhibited higher biofilm formation only than *K. variicola* (*p* < 0.05) (Fig. 1A). Similarly, in LB medium, *K. pneumoniae* formed significantly higher biofilm than *K. variicola* after 24, 48, and 72 h of incubation (*p* < 0.0001). After 48 and 72 h, *K. pneumoniae* exhibited significantly higher biofilm formation than *K. quasipneumoniae* (Fig. 1B).

### *K. quasipneumoniae* and *K. variicola* exhibited higher virulence to *Galleria mellonella* larvae compared to *K. pneumoniae*

**Fig. 2:**
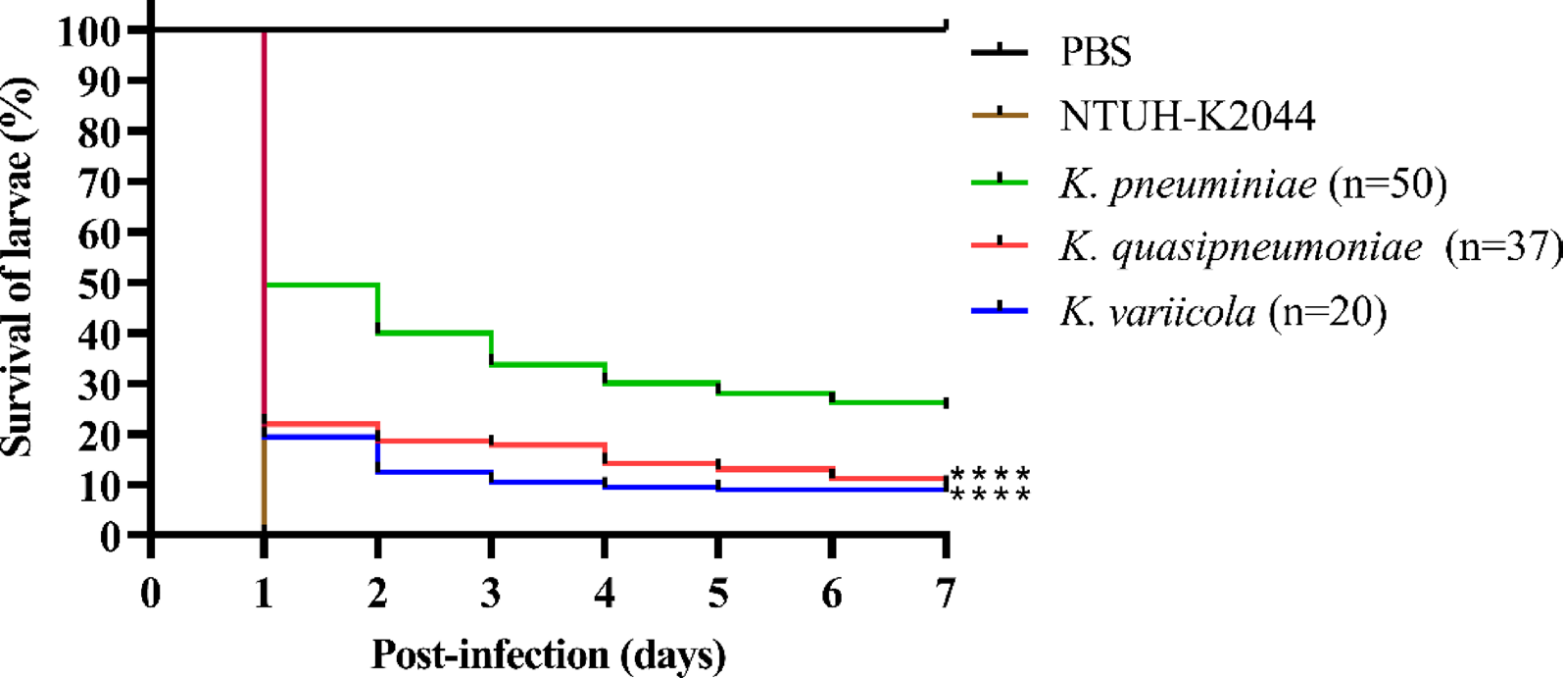
Observation of the seven-day mortality rate of larvae infected with *Kp*SC isolates of different species. The results are presented as the average survival proportion. Larvae injected with PBS were used as the negative control, whereas those injected with the hypervirulent *K. pneumoniae* isolate NTUH-K2044 served as the positive control in the experiment. Statistical comparisons were conducted using the *K. pneumoniae* group as the reference. ****, *p* < 0.0001

One day after infection with 2.5 × 10⁶ CFU of *K. pneumoniae*, *K. quasipneumoniae*, or *K. variicola*, larval survival proportions were 49.4%, 21.9%, and 19.4%, respectively (Fig. 2). On day seven, the survival rate for the *K. pneumoniae*-infected larvae remained at 25.1%, while only 9.7% and 8.5% of larvae survived in the *K. quasipneumoniae* and *K. variicola* groups, respectively (*p* < 0.0001) (Fig. 2). Consistent with previous studies, the hypervirulent *K. pneumoniae* isolate NTUH-K2044 exhibited high lethality toward the larvae, with all infected larvae dying within one day post-infection. In summary, our findings indicate that *K. pneumoniae* exhibits lower virulence toward larvae than *K. quasipneumoniae* and *K. variicola*.

## Discussion

In this study, we investigated the characteristics of *Kp*SC isolates in southern Taiwan for the first time and evaluated the genotypic and phenotypic differences among *K. pneumoniae*, *K. quasipneumoniae*, and *K. variicola*. MALDI-TOF is widely used for the rapid and accurate identification of bacterial species. Although MALDI-TOF cannot effectively identify *K. quasipneumoniae*, Rodrigues et al. reported that with improved database curation, MALDI-TOF MS could serve as a valuable tool for routine *Kp*SC identification [33]. Subsequently, Bridel et al. developed *Klebsiella* MALDI TypeR, a tool that utilizes new discriminant biomarkers in MALDI-TOF spectra to expand the number of identifiable *Klebsiella* species and improve the identification accuracy [20]. They reported an identification accuracy of 84%–100% depending on the phylogroup. In the present study, we primarily used multiplex PCR to screen isolates, followed by 16S rDNA sequencing and MALDI-TOF MS solely for cross-validation. This workflow ensures high accuracy in species identification while maintaining the efficiency of large-scale screening. Our findings demonstrated that multiplex PCR exhibited better specificity and sensitivity for *K. pneumoniae*, *K. quasipneumoniae*, and *K. variicola* identification in comparison with MALDI-TOF (Bruker Biotyper, version 2.0). However, identification methods for other species of *Kp*SC, such as Kp6 and Kp7, remain to be developed. Moreover, employing multiple complementary cross-validation methods can further enhance the accuracy of species identification. For instance, both *rpoB* phylogenetic analysis and average nucleotide identity (ANI) have been used to reveal a substantial number of misclassified *Klebsiella* spp. genomes originally reported as *K. pneumoniae* or *K. variicola* but later recognized as misidentified [34]. Nonetheless, a direct comparison of the accuracy among *rpoB* sequencing, 16S rDNA sequencing, MALDI TypeR, and multiplex PCR remains to be systematically evaluated in future studies.

Rodriguez-Medina et al. conducted a comprehensive analysis of 356 *Kp*SC isolates, identifying *K. pneumoniae* (95%, *n* = 338/356), *K. variicola* (2.5%, *n* = 9/356), and *K. quasipneumoniae* (2.5%, *n* = 9/356) [35]. Their findings align with our observed prevalence of *K. quasipneumoniae* and *K. variicola* within *Kp*SC, where among 809 *Kp*SC isolates, 4.6% were identified as *K. quasipneumoniae* and 2.4% as *K. variicola*. Moreover, 17 *K. quasipneumoniae* isolates were recovered from blood samples (45.9%), and 20 from urine samples, while 12 *K. variicola* isolates were from blood samples (60%) and 8 from urine samples (40%). These findings indicate that *K. variicola* may be more commonly isolated from blood samples. Potter et al. also reported important clinical ramifications, as high-risk antibiotic resistance genes are present in *K. variicola* genomes from a variety of geographic locations, and, as they demonstrated, *K. variicola* clinical isolates can establish higher bladder titers than *K. pneumoniae* [36]. However, due to the limited number of *K. quasipneumoniae* and *K. variicola* isolates identified in this study, we were unable to definitively determine the association between the prevalence of these species and the sample source or collection period. Therefore, the differences in the characteristics of *K. variicola*, *K. quasipneumoniae*, and *K. pneumoniae* from different specimen sources remain to be investigated. In addition, since all isolates in this study were obtained from a single medical center, future multicenter collections of *Kp*SC strains from different regions of Taiwan will be necessary to gain a more comprehensive understanding of their characteristics.

Capsule is considered the major virulence factor in *K. pneumoniae* and across the *Kp*SC. However, its extensive genetic diversity poses challenges for typification, particularly when relying on molecular K-typing schemes. While PCR-based approaches provide a rapid and relatively inexpensive method for determining the K locus, they cannot fully capture the true extent of capsule diversity. In line with this limitation, our study showed that 77.6% of the *Kp*SC isolates could not be assigned to any of the eight most common K-loci (K1, K2, K5, K20, K47, K54, K57, or K64) determined by PCR. Similarly, in our previous report analyzing 1,966 *Kp*SC isolates, 1,481 (75.3%) were not classified into these eight prevalent K-loci [26]. Moreover, *wzi* sequencing analysis of capsular types could not determine 18 out of 107 *Kp*SC strains (16.8%) in this study. Future studies will still need to incorporate *wzc* sequencing or even whole-genome sequencing to gain a more comprehensive understanding of the genomic characteristics of *Kp*SC [37].

We observed that all isolates that tested string test positive simultaneously carried both _*p*_*rmpA* and _*p*_*rmpA2*. However, three *K. pneumoniae* and 2 *K. quasipneumoniae* strains carrying _*p*_*rmpA* but testing negative in the string test, the likely explanation is genetic mutation. Yu et al. reported that DNA sequencing of the _*p*_*rmpA* and _*p*_*rmpA2* amplicons revealed indel mutations in the poly(G) tract within the _*p*_*rmpA* gene as well as _*p*_*rmpA2*, resulting in frameshift mutations in all non-hypermucoviscous isolates carrying _*p*_*rmpA* and/or _*p*_*rmpA2* [38]. In addition, mutations in the promoter region can reduce _*p*_*rmpA* expression, leading to diminished production of capsular polysaccharides and weakened virulence [39]. Therefore, although the isolates we identified carry _*p*_*rmpA* and _*p*_*rmpA2*, PCR alone cannot determine the exact gene sequence or expression level. These isolates may harbor mutations that result in string test-negative phenotypes and, consequently, might not be hypervirulent *K. pneumoniae*.

In previous epidemiological studies in Japan, *K. variicola* was more frequently isolated from older patients, while *K. quasipneumoniae* was more commonly isolated from patients with malignancy [6]. The mortality rates associated with clinical infections caused by these three pathogens also vary by region. In Stockholm, Sweden, *K. variicola* infection was associated with significantly higher mortality than *K. pneumoniae*, despite the absence of an apparent correlation with virulence factors such as capsule type [40]. In contrast, no significant differences in 30-day mortality rates were observed between the three species in Japan [6]. In this study, we observed that *K. pneumoniae* generally harbored a greater number of virulence-associated genes than *K. quasipneumoniae* and *K. variicola*. Consistently, *K. pneumoniae* isolates exhibited significantly stronger biofilm formation than *K. quasipneumoniae* and *K. variicola* in both LB and M9 media. Interestingly, despite stronger biofilm formation in *K. pneumoniae*, *K. quasipneumoniae* and *K. variicola* exhibited significantly higher lethality in the larvae infection model, suggesting that biofilm formation may not directly correlate with virulence in this model or that in vivo biofilm dynamics may differ from those observed under in vitro conditions. Moreover, three possible explanations may account for this observation: (1) differences in the in vivo expression levels of these virulence genes; (2) we only detected the presence of these genes, sequence variations such as single nucleotide polymorphisms (SNPs) may affect their functionality; and (3) the involvement of other virulence genes not targeted in this study, which may contribute to the higher virulence of *K. quasipneumoniae* and *K. variicola*. A limitation of this study is the relatively smaller number of *K. quasipneumoniae* and *K. variicola* isolates. Nevertheless, as indicated in Table 3, the prevalence of *kfuBC* was higher in *K. variicola*, which may partially explain their increased lethality in larvae. However, since this study is an epidemiological survey, we initially analyzed the correlation between the overall population’s virulence factor distribution and virulence in larvae. The individual strain-level differences within a single species were not further explored in this study. Future studies involving a larger collection of isolates or whole-genome sequencing analyses will be required to further identify virulence genes that differentiate *K. pneumoniae*, *K. quasipneumoniae*, and *K. variicola*. Previous studies have also indicated that although the larvae model is widely used to assess bacterial virulence [41], its results do not always correlate with those obtained from murine infection models. For example, it may not be reliably distinguishable between classical *K. pneumoniae* and hypervirulent *K. pneumoniae* strains [42]. Therefore, further investigation is needed to evaluate the pathogenicity of *K. pneumoniae*, *K. quasipneumoniae*, and *K. variicola* in mammalian hosts.

*K. variicola* and *K. quasipneumoniae* isolates have been reported to carry the *bla*_KPC_ and *bla*_NDM_ carbapenemase genes [43–46], which confer resistance to β-lactam antibiotics. Importantly, homologous recombination occurs among these three species [11]. The three pathogens also differ in their antimicrobial resistance profiles and the production of ESBLs. Among them, *K. variicola* exhibits resistance lower than that of *K. pneumoniae* and *K. quasipneumoniae* [47]. Our results demonstrated that *K. pneumoniae* exhibited higher resistance to gentamicin, sulfamethoxazole/trimethoprim, ciprofloxacin, and levofloxacin than *K. quasipneumoniae* and *K. variicola*. Additionally, the proportion of XDR isolates was elevated in *K. pneumoniae* compared to *K. quasipneumoniae* and *K. variicola*. These findings indicate that treatment options for *K. pneumoniae* clinical infections in Taiwan are more limited compared to *K. quasipneumoniae* and *K. variicola*. Recent studies have increasingly recognized the potential for plasmid transfer between *K. pneumoniae*, *K. quasipneumoniae*, and *K. variicola*, which contributes significantly to the dissemination of antibiotic resistance and virulence determinants [48–51]. Duran-Bedolla et al. showed that IncF plasmids were common in human *K. variicola* isolates but rare in plant isolates. Among the 297 incompatibility groups identified, IncFIBK, IncFIIK, and IncFIA/FIA(HI1) predominated, and these were linked to key resistance genes (e.g., *bla*_CTX−M−15_, *bla*_KPC−2_, *bla*_NDM−1_, and colistin resistance) and major sequence types (ST60, ST20, ST10) [52]. Recently, we reported for the first time the characteristics of carbapenem-resistant *K. quasipneumoniae* and carbapenem-resistant *K. variicola* isolates carrying carbapenemase genes in Taiwan, including conjugative metallo-β-lactamase-encoding plasmids such as IMP-8-MCR-9.1 in *K. quasipneumoniae* and VIM-1 in *K. variicola* [53]. These findings reveal the presence and potential spread of clinically relevant resistance determinants within Taiwanese *Kp*SC isolates and emphasize the need for continued surveillance. Further studies are warranted to collect additional clinical isolates for comprehensive genomic and phenotypic analysis and to monitor the horizontal transfer of virulence- and antibiotic resistance-associated plasmids among these species.

## Conclusion

This study is the first to report the distribution of *K. pneumoniae*, *K. quasipneumoniae*, and *K. variicola* isolates in Taiwan, highlighting differences in virulence-associated gene profiles, antimicrobial resistance, and biofilm formation capacities. Notably, *K. quasipneumoniae* and *K. variicola* exhibited higher virulence than *K. pneumoniae* in the larvae infection model. The potential for horizontal transfer of antimicrobial resistance and virulence plasmids among members of the *Kp*SC, as well as the possibility of genome recombination, underscores the need for ongoing surveillance.

## Data Availability

The other data will be available on request.
